# Metasurface based on phase change materials for electrically reconfigurable THz beam steering in copolarized transmission mode

**DOI:** 10.1038/s41598-025-11713-4

**Published:** 2025-11-19

**Authors:** Krishna Kumar, Borja Vidal, Carlos Garcia-Meca

**Affiliations:** 1 DAS Photonics, 46022 Valencia, Spain; 2 Nanophotonics Technology Center, Universitat Politecnica de Valencia, 46022 Valencia, Spain; 3 Monodon, Navantia, 28005 Madrid, Spain

**Keywords:** Phase change metasurfaces, Split ring resonator, Beam steering, Inverse design, Tunable phase modulator, Metamaterials, Photonic devices, Terahertz optics, Phase transitions and critical phenomena, Sub-wavelength optics

## Abstract

Metasurfaces are an attractive technology to develop electronically-controlled beam steering devices of THz waves. However, the dynamic steering of co-polarized transmitted waves at a fixed frequency has not been demonstrated yet using this scheme. The performance of this configuration is usually limited by the low phase shifts achievable from thin-film reconfigurable meta-atoms, necessitating the exploration of novel methods. Here, we propose an alternative approach to address this challenge by designing a tunable metasurface utilizing a phase modulation range of only 116$^{\circ }$. Our design employs a meta-atom array incorporating VO$_2$ patches within C-shaped split ring resonators (CSRRs). The metal-to-insulator transition of VO$_2$ enables a continuously tunable phase shift with a reduced amplitude modulation, resulting in a dynamic control over the direction of co-polarized transmitted beams at 0.75 THz in the angular range spanning from -56$^{\circ }$ to +56$^{\circ }$. Furthermore, we enhance the device performance by co-optimizing the distribution of phase and amplitude of the gradient profile, leading to an increase in transmission efficiency. This approach can be extended to other regions of the electromagnetic spectrum, accessing applications that require tunable beam steering operation such as imaging, LIDAR, and 6G telecommunications that cannot achieve a 360$^{\circ }$ phase modulation range.

## Introduction

THz technology has found several applications in the field of wireless communication^1,2^, industrial quality control^3,4^, biomedical imaging^5^, and spectroscopy^6,7^. However, harnessing its full potential requires precise control over the phase^8^, amplitude^8^ and polarization^9,10^ of THz waves. Metasurfaces and metagratings provide unprecedented control over electromagnetic waves by allowing their modulation on a sub-wavelength scale using appropriately designed meta-atom resonators^11^. Several methods employing metasurfaces^12^ and metagratings^13,14^ have been reported for the full control of THz radiation. For instance, one of the fundamental functionalities in THz photonics, namely, beam steering, which we will focus on in this paper, has been demonstrated using split ring resonators (SRRs)^15,16^. However, such metasurfaces are composed of passive components, limiting their operation to fixed configurations once fabricated. This constraint results in devices that cannot change the steering angle for a given frequency (the steering angle might be tuned at the cost of changing the frequency, an effect usually referred to as beam squinting). Therefore, integrating active elements is essential to implement tunable operations at a fixed frequency^9,17^.

Various techniques have been proposed to achieve reconfigurable metasurfaces^18–20^ for THz dynamic beam steering^21^. For instance, experimental demonstrations in transmission mode based on liquid crystals have been reported^22,23^, although this technology presents high losses and low switching times. Probably, the approach that provides the optimal combination of switching speed and large variation of the properties of the meta-atom relies on the integration of phase change materials (PCMs)^24^, such as Ge$_2$Sb$_2$Te$_5$ ^17^ and VO$_2$ ^25,26^. These materials have an ultra-fast response^27^ to external stimuli, including thermal^25,28^, electrical^29,30^ and optical^31–33^ excitation. They exhibit a metallic-to-insulator transition (MIT) that results in tunable optical properties with large optical contrast, a highly desirable feature for reconfigurable metasurfaces. Consequently, a simple strategy to transform passive metasurfaces into active ones relies on adding a thin layer of a PCM^17,25^. However, such a layer typically introduces losses upon state transitions resulting in a non-ideal optical behavior, such as having a co-varying phase and scattering amplitude^34–36^, limiting its application as a beam steering device, where constant amplitude and large phase modulation are desirable^11^. Actually, reaching a full 360$^{\circ }$ phase modulation range while keeping the amplitude approximately constant with reconfigurable meta-atoms is extremely difficult^37^. Working in transmission mode instead of with reflective metasurfaces is also convenient in various applications, e.g., when the source is close to the metasurface and can block the reflected wave. Finally, in many situations, the metasurface should also conserve the polarization.

A few theoretical studies seeking to achieve some of the mentioned properties have been reported. Graphene metasurfaces are an example, although 360$^\circ$ phase modulation was only obtained for reflective metasurfaces^7^, while for devices working in transmission mode, phase tuning was obtained by changing the geometry of the meta-atom in a non-reconfigurable way^9^. Another strategy for building transmission-mode reconfigurable metasurfaces involves integrating small patches of the reconfigurable material in the meta-atom (instead of thin films), although with a maximum reported phase shift of 150$^\circ$ accompanied by a considerable amplitude variation, with which beam steering was not demonstrated^35,38^. Furthermore, these metasurfaces were either optically controlled (usually, electrically-reconfigurable devices are more practical and compact) or necessitated complex fabrication mechanisms. Finally, a recent approach smartly incorporates several VO$_2$ patches per meta-atom, which can be turned on or off independently to open or close gaps in an SRR. This mitigates the losses and achieves a 360$^{\circ }$ phase modulation range^26^. However, this approach only allows a discrete number of phase shift values per unit cell and requires many control units to implement the steering operation. Moreover, this metasurface only works for the cross-polarized transmitted beam. In summary, none of the reported proposals enables a rapidly and electrically reconfigurable beam steering functionality in co-polarized transmission mode at a fixed frequency, even less permitting a continuously variable 360$^\circ$ phase modulation range.

Remarkably, some studies suggest that, even with a reduced phase modulation range, it is possible to achieve a good beam steering performance^25,39–41^. For example, conventional coding metasurfaces rely on a binary approach, utilizing only one-bit phase shifts (0$^{\circ }$ and 180$^{\circ }$) to demonstrate beam steering^41–44^. However, as far as we know, the reported coding metasurfaces only operate in reflection mode. Additionally, they are prone to generating undesired scattering lobes that are more intense than the desired beam^41,43^. In contrast, the inverse design method is significantly more effective than coding metasurfaces when the phase modulation range is significantly lower than 360$^{\circ }$. This technique leads to reduced side lobes and enables efficient beam steering by co-optimizing the amplitude and phase distribution of the meta-atom arrays. Moreover, the inverse design method offers the flexibility to enhance specific characteristics of the beam, such as the directivity and the transmission efficiency of the main scattering lobe^39^. Nonetheless, up to date this approach has not been shown to work at THz frequencies nor in transmission mode and has not employed PCMs as the reconfigurable material.

Here we propose a reconfigurable metasurface based on SRRs and VO$_2$ patches that works in co-polarized transmission mode and that can steer a beam at an angle that can be dynamically controlled using an electronic device, such as a field-programmable gate array (FPGA). Specifically, the metasurface has an electrically tunable period, with which we can select the allowed diffraction orders. Moreover, the phase delay introduced by the meta-atoms within each period can be changed continuously and chosen to favor the transmission along a single desired diffraction order. In addition, we co-optimized the distribution of phase and amplitude of the meta-atoms in the metasurface to improve the radiation efficiency of the transmitted beam. The proposed metasurface can be designed using patch resonators beyond SRRs.

## Results

### Structure and theoretical analysis of the metasurface

**Fig. 1:**
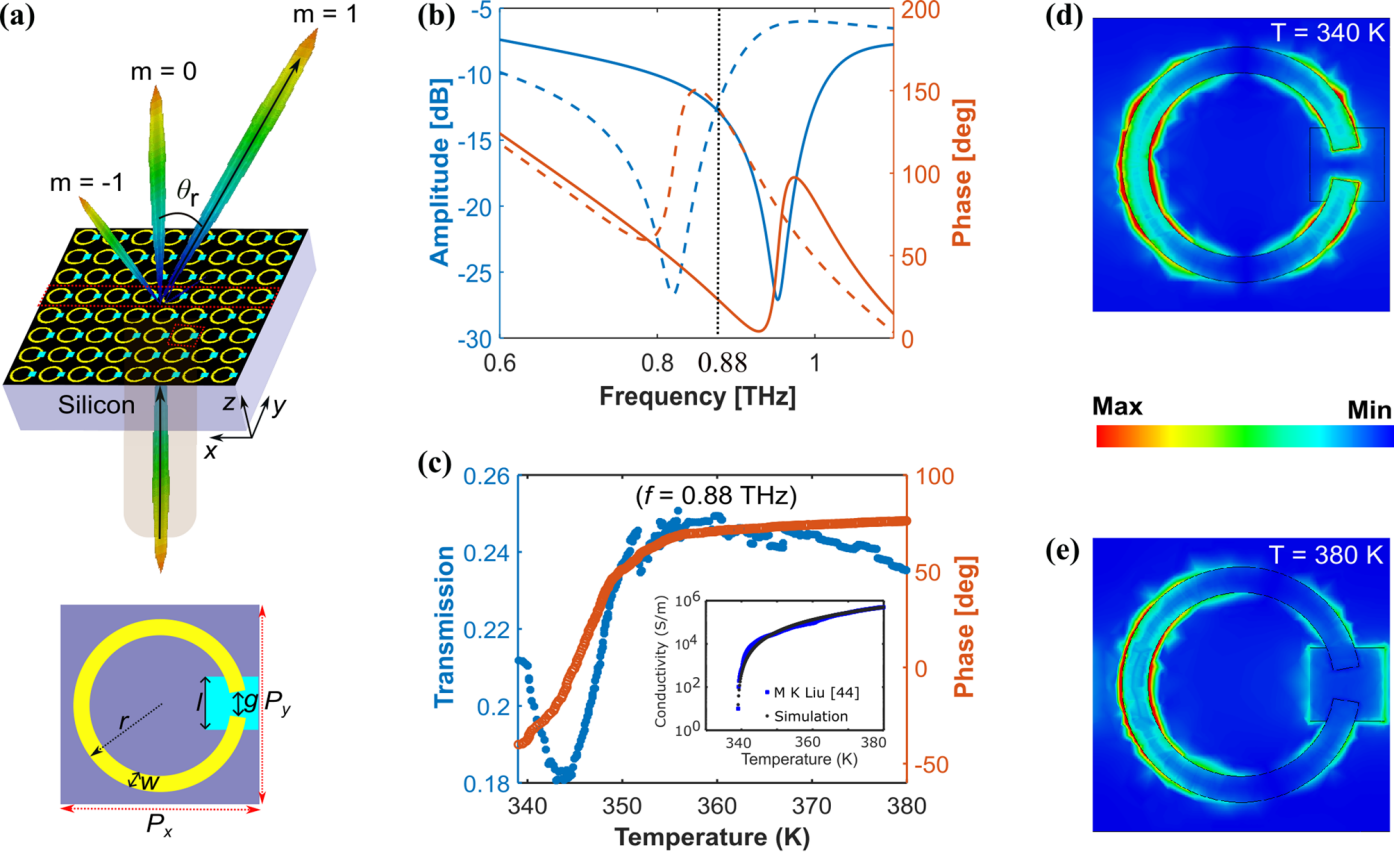
(**a**) Schematic of the metasurface. The optimized parameters of the meta-atom are $P_x$ = 80 $\mu$m, $P_y$ = 80 $\mu$m, *l* = 20 $\mu$m, *w* = 7 $\mu$m, *g* = 10 $\mu$m, $r = 32$ $\mu$m. The spatial modulation of the conductivity of the VO$_2$ patches in a supercell (illustrated within the red dashed box) induces a phase gradient resulting in a beam steering along $\theta _r$. An anomalously transmitted beam is also excited at -$\theta _r$ (corresponding to $m = -1$). (**b**) Transmission amplitude and phase of the metasurface when all the meta-atoms are in the same state. The dashed line corresponds to the metallic state of VO$_2$, while the solid line corresponds to the insulating state of VO$_2$, (**c**) Simulated transmission amplitude and phase of the meta-atom as a function of *T* at $f=$ 0.88 THz. The inset includes a plot of the experimentally measured conductivity of a 300 nm VO$_2$ thin film at varying temperature taken from^48^, alongside a comparison with the conductivity data used for simulations. (**d-e**) Electric field distributions on the surface of the meta-atom for the (**d**) insulating and (**e**) metallic state of VO$_2$.

The proposed metasurface is designed to operate for *x*-polarized incident beams and consists of a periodic array of meta-atoms, each comprising a 20-$\mu$m-thick silicon substrate, a 200-nm-thick Al C-shaped SRR (CSRR), and a 300-nm-thick VO$_2$ patch positioned near the CSRR gap and over the substrate (see Fig. 1a). Compared to traditional beam steering mechanisms, which often employ thin layers of PCMs, patches introduce lower losses while offering an equivalent phase modulation range. Additionally, heating a smaller patch is more energy-efficient and logistically simpler than uniformly heating a thin film of PCM. Joule heating via microheaters integrated near the VO$_2$ patch can be used to locally heat the VO$_2$. For example, electrodes made up of metals such as Ti can be placed at the edges of the VO$_2$ patch to create a uniform current distribution, thereby inducing localized heating and triggering the phase transition in VO$_2$. An FPGA can be utilized to control the electrical connections to the electrodes and microheaters through wire bonding, ensuring reliable and precise control over the heating process, and thus over the phase transition in VO$_2$. Moreover, employing VO$_2$ has the additional advantage that its transition temperature is lower compared to other PCMs, making it highly convenient for active photonic devices. The temperature-dependent complex permittivity of VO$_2$ in the THz region can be described using a Drude model^45^:

1 $$\begin{aligned} \varepsilon (\omega , T) = \varepsilon _\infty - \frac{\omega _p^2(T)}{{\omega ^2 + i\omega _d\omega }} \end{aligned}$$

Here, *T* is the temperature, $\omega = 2\pi f$ (*f* being the frequency), $\varepsilon _\infty$ = 12 is the high-frequency permittivity, $\omega _d$ = $5.75 \times 10^{13}$ s$^{-1}$ is the collision frequency and $\omega _p$ = $(\sigma (T)\omega _p^2(\sigma _0)/\sigma _0)^{1/2}$ is the effective plasma frequency, where $\sigma (T)$ represents the temperature-dependent conductivity, with $\sigma _0$ = $3 \times 10^{5}$ corresponding to the conductivity at a plasma frequency of $\omega _p(\sigma _0)$ = $1.4 \times 10^{15}$ s$^{-1}$. Along the MIT, $\sigma (T)$ varies from 10 S/m^36,46^ to $5 \times 10^5$ S/m^30,47^. According to experimental data, this corresponds to a temperature variation from 339 K to 380 K^48^. The measured temperature-conductivity curve is shown in the inset of Fig. 1c, which perfectly matches the relation used in our simulations. The electromagnetic response of the meta-atom at different states of VO$_2$ was studied in CST Studio considering periodic boundary conditions along the *x*- and *y*-directions and open boundary conditions along the *z*-direction. As is well known, the CSRR behaves as a resonant structure that manifests as a dip in the transmission spectrum (see Fig. 1b). The resonant frequency of the CSRR depends on the conductivity of VO$_2$, which can be controlled through an electronic circuit made up of transparent electrodes. In particular, the CSRR becomes a closed circuit at $\sigma = 5 \times 10^5$ S/m (metallic state of VO$_2$) with an associated resonant frequency of 0.82 THz. As $\sigma$ decreases, the resonance gradually blue-shifts up to 0.953 THz at $\sigma = 10$ S/m (insulating state of VO$_2$), for which the CSRR behaves as an open circuit^48–51^. The electric field distribution of the open and closed circuit condition is depicted in Fig. 1d and e. This frequency variation results in a tunable phase with a modulation range of 116$^{\circ }$ at 0.88 THz (see Fig. 1c). We also studied the amplitude modulation range of the meta-atom within this frequency range, defined as:

2 $$\begin{aligned} A_{\text {mod}} = \frac{{(A_{\text {max}})^2 - (A_{\text {min}})^2}}{{(A_{\text {min}})^2}} \end{aligned}$$

where $A_{\text {max}}$ and $A_{\text {min}}$ are the maximum and minimum transmission amplitude of the meta-atom in the previous frequency range (see Fig. 1c). We obtained a very low value of 0.9267 for $A_{\text {mod}}$ as is desired.

For a normally-impinging incident wave, the angles $\theta _m$ associated with the diffraction orders allowed by the metasurface^39,40^ are governed by the grating equation^52^:

3 $$\begin{aligned} \theta _m = \sin ^{-1}\left( m\frac{\lambda }{n L}\right) \end{aligned}$$

where *n* is the refractive index of the background, *m* is the diffraction order, and *L* is the grating period. This period can be dynamically controlled by varying the phase of each meta-atom to build a periodic phase function and, consequently, it is an integer multiple of the meta-atom width. Therefore, the first step to build a beam steering device is to select *L* so that $\theta _m$ coincides with the desired steering direction for some *m*, typically $m = 1$. In addition, the excitation amplitude of each diffraction order is determined by the phase delays introduced by the meta-atoms within one period, which we can optimize to maximize the amplitude associated with $\theta _r = \theta _{1}$ and to minimize the amplitude of the rest of the grating orders. When a complete 360$^\circ$ modulation range is available, this is usually accomplished by implementing a varying phase profile $\phi (x)$ that fulfils the generalized Snell’s law:

4 $$\begin{aligned} \sin (\theta _r) = \frac{\lambda }{2\pi n}\frac{d\phi (x)}{dx} \end{aligned}$$

**Fig. 2:**
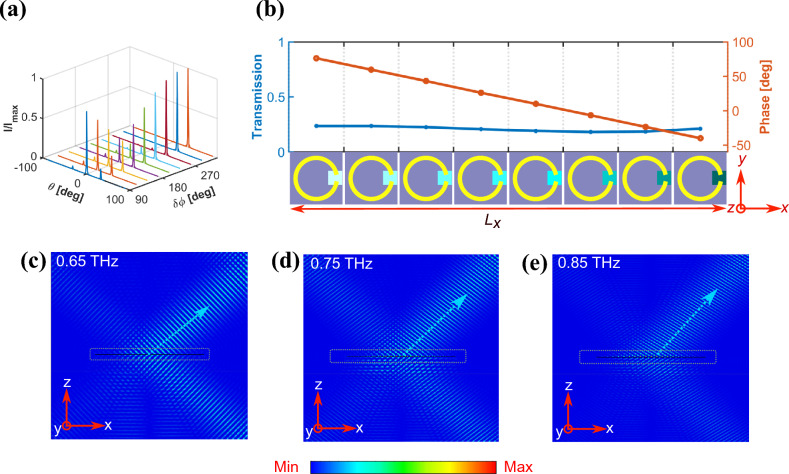
(**a**) Normalized intensity of an ideal phase modulated metasurface. The far-field intensity is analytically calculated for a 1D phase gradient comprising 168 meta-atoms that repeats each 8 meta-atoms. Mutual coupling is neglected to reduce the calculation complexity by assuming meta-atoms as omnidirectional scatterers. (b) Illustration of a linearly decreasing phase-gradient profile, designed for beam steering at a frequency of 0.88 THz. (c)-(e) Simulated electric field distribution for the device in (**b**) at various discrete frequencies. A periodic array of the phase-gradient comprising 168 meta-atoms is placed along the *x*-axis (within the red dotted square box). For clarity, the electric field associated with the normally incident, reflected, and transmitted beams has been subtracted.

For the metasurface, *dx* can be taken as the width of the meta-atom and $d\phi$ as the phase shift between neighbouring meta-atoms. However, when a limited modulation range is available, as in our case, this relation does not provide, in general, the optimum phase profile $\phi (x)$ for the beam steering functionality. The effect of the phase modulation range $\delta \phi$ on the far-field scattering pattern of an ideal linear meta-atom array has been analytically studied, using the far-field intensity:

5 $$\begin{aligned} I(\theta ,f) = I_\textrm{antenna}(\theta ,f)\left| \sum A_n(T_n, {f}) \exp (i\phi _n(T_n, {f})) \exp (ik(n-1)P_x \sin \theta ) \right| ^2 \end{aligned}$$

where $k=2\pi /\lambda$, $I_\textrm{antenna}(\theta ,f)$ is the far-field intensity of a single antenna (meta-atom in our case), the sum inside the bars is the antenna array factor (AF), and $T_n$ is the temperature that controls the amplitude $A_n$ and phase $\phi _n$ of the *n*-th antenna. For this study, we considered 300 meta-atoms with a constant transmission amplitude and neglected the potential mutual coupling effect between meta-atoms to reduce the calculation complexity. In Fig. 2a, the effect of a limited value of $\delta \phi < 360^\circ$ was studied, showing that it can significantly affect the far-field radiation pattern. When the phase modulation range reaches nearly 150$^{\circ }$, the intensity of the $m=0$ transmitted beam and the desired steered beam ($m=1$) became comparable at the design frequency of 0.88 THz. While we only obtain a value of $\delta \phi = 116^\circ$, we will see that, after some additional optimization of the configuration and working frequency, it will allow us to achieve a zeroth order intensity significantly lower than that of the $m=1$ order.

### Spectrally resolved beam steering

A schematic of the proposed design is presented in Fig. 2b. To validate our theoretical assumptions, we conducted finite difference time domain (FDTD) quasi-2D simulation using CST Studio. Exploiting the transmission and phase characteristics of the proposed meta-atom, we designed an array with a linearly decreasing phase-gradient profile with a period of 8 meta-atoms, and each meta-atom having a different value of $\omega _p$ to cover the available phase modulation range of 116$^{\circ }$. Subsequently, the designed linear array, comprising 168 meta-atoms, was placed along the *x*-axis and illuminated with an *x*-polarized Gaussian source of width $P_y$ and a beam radius of 10$\lambda$, where $\lambda$ is the wavelength corresponding to the operating frequency of 0.88 THz. Boundary conditions were considered open along the *x*-axis, periodic along the *y*-axis, and open along the *z*-axis. The reason to select a number of 168 meta-atoms is to accommodate the size of the *x*-polarized incident Gaussian beam of radius 10$\lambda$.

When the VO$_2$ of all meta-atoms remains in its dielectric state at room temperature, the metasurface exhibits no phase gradient, causing straightforward transmission of the normally incident *x*-polarized Gaussian beam. Upon imparting the aforementioned linearly decreasing phase profile by selectively controlling the conductivity of each meta-atom, the transmitted beam undergoes deflection along the first-order diffraction direction. The simulated electric field distribution of the device depicted in Fig. 2b is shown in Fig. 2c, d, and e for three different frequencies. It is worth noting that, although the characteristic distance, $(2P_x^2)/\lambda \approx 37~\mu \text {m}$, which is required to avoid near-field coupling between neighboring meta-atoms, is smaller than the interspacing $P_x = 80~\mu \text {m}$ between meta-atoms, the separation between the C-shaped resonant structures ($\approx 16~\mu \text {m}$) is not as evident from Fig. 2b. This results in near-field coupling between adjacent meta-atoms, leading to undesired electromagnetic interference effects. These effects become more pronounced when the VO_2_ patches are in their metallic state (refer to Fig. 1d and e). In this state, the characteristic distance between the resonant structures is further reduced, increasing the electromagnetic crosstalk. Thus, the strength of this coupling appears to be strongly influenced by the crystallinity of VO_2_: minimal in the insulating state, maximal in the metallic state, and intermediate when VO_2_ is in a mixed state (comprising both the insulating and the metallic domains). The metaatoms that form the phase gradient, as shown in Fig. 2b, consist of VO_2_ in all three states: insulating, metallic, and intermediate. This variation introduces an overall coupling effect that causes the actual phase and amplitude profiles of the array to deviate from the intended design.

**Fig. 3:**
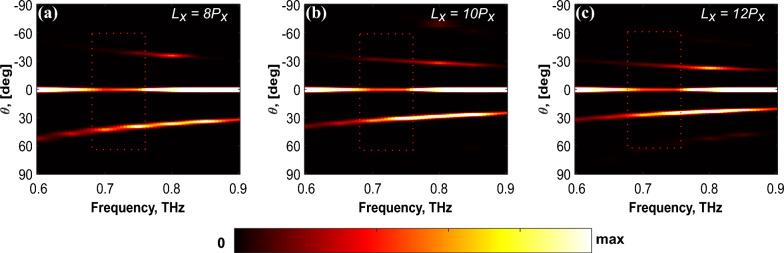
Transmission intensity spectrum of the three metasurfaces of phase-gradient length (**a**) 8$P_x$ (**b**) 10$P_x$, and (**c**) 12$P_x$. As evident from the red dashed box in each case, the frequency bandwidth is limited to the band spanning from 0.7 THz to 0.76 THz.

As a result, a noticeable red shift in the operational frequency is observed, leading to an optimal performance at 0.75 THz, as depicted in Fig. 3. The optimal range for effective beam steering, characterized by a higher intensity of the desired steered beam compared to undesired diffraction orders, is confined to the frequency band from 0.7 THz to 0.76 THz. As can be seen from Fig. 3, this performance remains consistent across different configurations of variable periodic length phase-gradient metasurfaces.

### Reconfigurable metasurface for dynamically-tunable beam steering

**Fig. 4:**
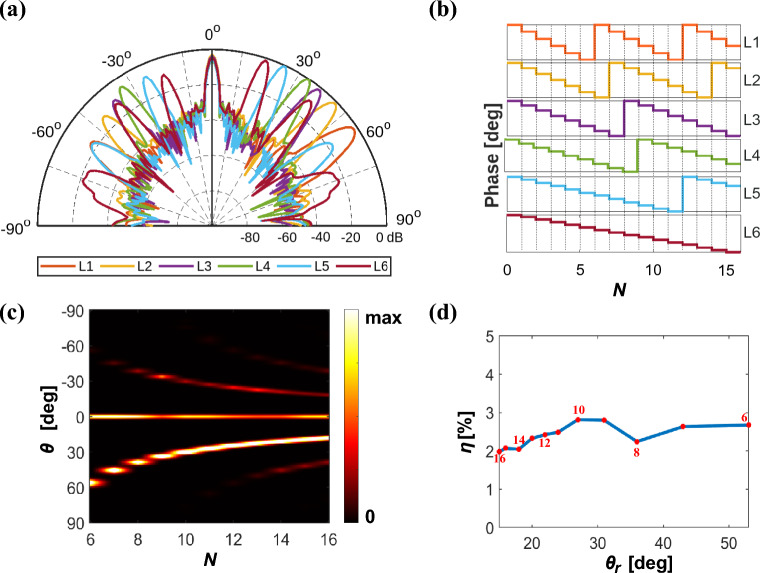
(**a**) Normalized far-field intensity of 6 metasurfaces and (**b**) their corresponding phase gradient profile. (**c**) Transmission intensity of 11 metasurfaces as a function of $L_x$ in the angular range of -90$^{\circ }$ to +90$^{\circ }$, demonstrating beam steering from 18$^{\circ }$ to 56$^{\circ }$. (d) Transmission efficiency of the 11 metasurfaces.

By spatially controlling the value of $\omega _p$ of each meta-atom, a metasurface with a varying phase-gradient and grating period ($L_x$) has been created. The length of $L_x$ is used to control the angle of diffraction and the slope for deciding the order of diffraction along which the beam will be diffracted. According to equation 3, the scattering angle $\theta _r$ can be dynamically adjusted by varying the length of the grating period $L_x$ at a fixed $\lambda$. However, $L_x = N P_x$ is proportional to the number of meta-atoms (*N*) in a period and to the meta-atom width ($P_x$), the latter being fixed in the metasurface, which results in a discrete nature for $L_x$. While this limitation imposes a discretization of the steering angle, carefully distributing the phase-shifts along the meta-atom array enables many steering directions. To demonstrate this, we designed six linear arrays of meta-atoms, each composed of 168 meta-atoms with varying lateral grating periods (*L*1 to *L*6 as shown in Fig. 4). These metasurfaces were illuminated with the CST built-in Gaussian beam source whose width was selected to be equal to $P_y$. We used periodic boundary conditions along the *y*-axis and open boundary conditions along both the *x*-axis and the *z*-axis. The far-field radiation pattern of the arrays and its corresponding phase gradient profile is presented in Fig. 4. The simulated steering angle $\theta _r$ agrees well with the theoretically calculated angle $\theta _{theo}$ as listed in Table 1. We observed that when the lateral grating period is between $\lambda$ and $2\lambda$, the transmitted wave primarily exhibits a first-order diffraction with higher transmission efficiency concentrated in the main lobe (Fig. 4, *L*1, *L*2, *L*3, *L*4). Conversely, larger grating periods lead to the appearance of high-order diffraction modes and reduced transmission efficiency (Fig. 4, *L*5, *L*6). Nevertheless, a constant transmission efficiency was obtained over a broad range of 18$^{\circ }$ to 56$^{\circ }$. Further, we designed additional metasurfaces to cover a spectrum of grating lengths from 6$P_x$ to 16$P_x$. The normalized transmission intensity as a function of $L_x$ for 11 metasurfaces is presented in Fig. 4c, ensuring a grating length greater than the width of 5 meta-atoms for optimal performance^52^. The steering range can be further increased to other quadrant by reversing the phase distribution, resulting in a broad beam steering range of -56$^{\circ }$ to +56$^{\circ }$. In addition, we studied the angular width of the steered beam by calculating its full width at half maximum (FWHM) as listed in Table 1. As expected, we obtained decreasing values of FWHM for increasing *N*, with a maximum of 6.43$^\circ$ observed for $N = 6$. To further assess the performance of the steering device, we calculated the transmitted radiation efficiency $\eta _\textrm{rad}$ at $\theta _r$ using the expression:

6 $$\begin{aligned} \eta _\textrm{rad}(\theta _r) = \frac{I(\theta _r) \times \textrm{FWHM}(\theta _r) \times 100}{I_\textrm{rad}} \end{aligned}$$

where $I(\theta _r)$ is the transmission intensity at $\theta _r$, and $I_\textrm{rad}$ is the integral of the intensity scattered over the full angular range from 0 to $360^{\circ }$. The calculated efficiencies are listed in Table 1 and shown in Fig. 4d. We obtained almost a constant transmission efficiency in a broad angular range, from 18$^{\circ }$ to 56$^{\circ }$, making it suitable for several applications where constant power throughout the steering range is required. The radiation efficiencies of the steered beam can be further optimized with the help of phase profile optimization, such as inverse design optimization (see below). By co-optimizing the distribution of phase profile along the array, it is possible to further suppress the unwanted scattered lobes. By doing so, we obtained a maximum transmission efficiency of 3.98% at a steering angle of 25$^{\circ }$. Note that this efficiency is higher than that obtained in previous works (in Table 2 we show a comparison of different features of our work and previous beam steering metasurfaces with limited phase modulation range $\delta \phi$). On the other hand, it might be further improved by exploiting hybrid resonant structures (e.g., C-shaped structures combined with dipole resonators) that support strongly localized coupling modes. Another option could be to integrate these structures with low-loss phase change materials in place of VO$_2$. For example, materials such as Ge$_3$Sb$_2$Te$_6$^53,54^, Ge$_1$Sb$_2$Te$_4$^55^, Ge$_1$Sb$_4$Te$_7$^55^, Sb$_2$Se$_3$^56,57^, and Sb$_2$S$_3$^56,58^ have demonstrated lower optical losses in the visible and near-IR regions but have not been applied in the THz region. However, the fact that these devices have memory complicates the switching operation from the crystalline state to the amorphous state, as this requires rapid quenching, which is challenging given the large material volume demanded to implement THz resonators. Moreover, optimizing the phase and amplitude distribution using hybrid models (genetic algorithms and gradient-based optimization) may play a role in further improving the efficiency of the device. In applications for which the power budget is critical, low power efficiency could be a limiting factor. However, the value we obtain can be acceptable for applications in which the critical parameter is the radiation pattern.

**Table 1:** Transmission efficiency, $\eta$, and bandwidth, FWHM, of 11 metasurfaces operating at a frequency of 0.75 THz.

$L_x (\times P_x)$	6	7	8	9	10	11	12	13	14	15	16
$\theta _r$ (deg)	56	45.6	38.7	33.7	30	27	24.6	22.6	20.9	19.4	18.17
$\theta _{theo}$ (deg)	56.44	45.58	38.68	33.74	30	27.03	24.62	22.61	20.92	19.47	18.21
FWHM (deg)	6.43	5.31	4.7	4.3	4.17	4.15	4.03	3.97	3.89	4.02	3.89
$\eta (\%)$	2.67	2.63	2.24	2.80	2.81	2.48	2.42	2.32	2.04	2.06	1.97

**Table 2:** Comparison of different active beam steering metasurfaces with limited phase modulation range. ’C’ and ’D’ denote continuous and discrete phase modulation, respectively. ITO stands for indium tin oxide. ’R’ and ’T’ indicate reflection and transmission modes, respectively. $*$ denotes the findings of this work.

Ref	$\lambda$ / *f*	$\delta \phi$	Efficiency	$$\begin{array}{l}{Active} \\ {element} \end{array}$$	Mode	Comments
^59^	1510 nm	C: $270^\circ$	2.2%	ITO	R	No analogous design in the THz range
^39^	1510 nm	C: 272$^\circ$	2.7%	ITO	R	No analogous design in the THz range
^36^	0.1 THz	C: 57$^\circ$	Not reported	VO_2_	T	$$\begin{array}{l}\text {Very low } \delta \phi \text {; Device size} \approx \lambda \rightarrow \text {Low directivity} \\ \text {and strongly limited incident beam size} \end{array}$$
^26^	0.6 THz	D	Not reported	VO_2_	T	$$\begin{array}{l}\text {Requires individual control of 8 VO}_2 \text { elements} \\ \text {per meta-atom; Limited angular resolution;} \\ \text {Cross-polarized mode} \end{array}$$
^41^	0.22 THz	D	Not reported	VO_2_	R	Limited angular resolution
^42^	0.4 THz	D	Not reported	VO_2_	R	$$\begin{array}{l}\text {Requires individual control of 3 VO}_2 \text { elements} \\ \text {per meta-atom; Limited angular resolution} \end{array}$$
$*$	0.75 THz	C: 116$^\circ$	3.98%	VO_2_	T	This work

### Effect of repetition number

**Fig. 5:**
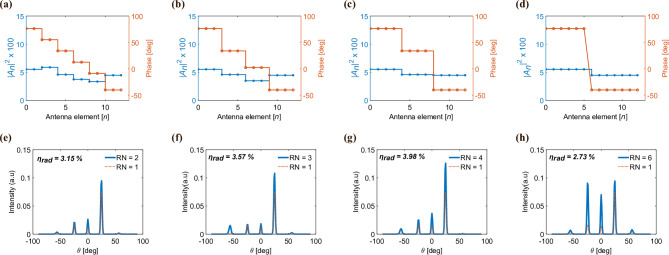
Simulated far-field intensity of two-level, three-level, four-level, six-level, and twelve-level phase profiles with repeating meta-atom phases corresponding to repetition numbers RN = 6, 4, 3, 2, and 1. The corresponding transmission efficiencies are 2.73$\%$, 3.98$\%$, 3.57$\%$, 3.15$\%$, and 2.42$\%$

Taking into account the exponential variations in the conductivity of VO$_2$ near its MIT transition, experimentally achieving a large number of phase points across this transition may be challenging. For instance, the phase-shift generally depends on how precisely one can control the applied currents in an electronically controlled VO$_2$-based meta-atom. Hence, it becomes important to optimize the phase profile to function effectively with minimal phase points. To address this challenge, we have implemented a methodology wherein the length of the grating period is conserved while optimizing the phase profile. As illustrated in Fig. 5, we have introduced a repeated state for each RN (repetition number) adjacent meta-atoms within each linear phase gradient supercell. Each of these supercells comprises 12 meta-atoms, resulting in phase gradients with stair-step profiles of 2 (Fig. 5d and 5h), 3 (Fig. 5c and 5g), 4 (Fig. 5b and f), 6 (Fig. 5a and e), and 12 levels, with 12 representing the forward-designed metasurface. Through quasi-2D simulations, we have investigated the corresponding far-field intensity patterns. As can be seen from Fig. 5b, d, f, and h, the maximum intensity is observed for the 3-level stairstep phase profile, with each level consisting of 4 meta-atoms in the same VO$_2$-state (RN = 4). Although there is a slight enhancement in side lobes observed for RN = 2, 3, and 4 compared to the forward-designed scenario (RN = 12), the increase in the intensity along the main steering direction favors the RN approach over the forward designed beam-steering device. Furthermore, the phase pattern depicted in Fig. 5h bears resemblance to 1-bit coding metasurfaces, wherein 0 denotes the minimum phase value and 1 indicates the maximum phase values. Nevertheless, the far-field intensity pattern of 2-level stair-step phase profiles exhibits similarities with the recently proposed 1-bit coding metasurfaces by Daquan^41^, suggesting that solely relying on the purely insulating and metallic states of VO$_2$, may not always yield optimal results. Hence, it is important to attain maximum phase points for effective beam steering applications. To further support our results, we compared the transmission efficiency of the main lobe for each value of RN. As can be seen in Fig. 5g, the efficiency for RN = 4 is higher than the forward-designed phase-gradient profile. This kind of optimization based on repeated meta-atoms can be applied to any value of *N* that is not a prime number.

### Inverse design optimization

Another method employed to enhance the beam steering performance is the inverse design optimization technique. Along this line, we utilized a genetic algorithm (GA) in MATLAB to optimize the objective figure of merit, expressed as

7 $$\begin{aligned} \text {FOM}(\textbf{X}_i, {f}) = D_{\text {ideal}}(\theta _r, {f}) - D(\theta _r, {f}) \end{aligned}$$

Here, $D_\textrm{ideal}(\theta _r,f)$ is the target directivity and $D(\theta _r, {f})$ is the directivity of the steered beam at a given frequency *f*. The directivity is calculated analytically using the antenna array factor of equation 5 as $|\textrm{AF}|^2$ (assuming approximately omnidirectional antennas). The optimization parameter is the index vector $\textbf{X}_i$, which contains *i* positions of a list of possible meta-atom configurations (*i* defines the number of meta-atoms of the super-cell, which is repeated periodically to form a 1D array of 168 meta-atoms). Each position *n* of the list is linked to the amplitude $A_n(T_n,f)$ and phase $\phi _n(T_n,f)$ response values of one of 300 possible meta-atom configurations, each corresponding to a different temperature $T_n$. The values of $A_n(T_n,f)$ and $\phi _n(T_n,f)$ are derived from CST Studio simulations at $f = 0.88$ THz (refer to Fig. 1c).

To optimize the FOM, the GA iteratively adjusts the phase and amplitude distribution of the meta-atoms. The algorithm considers initial indices $\textbf{X}_i$, corresponding to specific meta-atom configurations within the super-cell (e.g., for $i = 8$ we have 8 indices: $\textbf{X}_8$ = (1,7,55,90,105,155,265,300), each associated with a specific meta-atom configuration), and repeats these periodically to create a 1D array of 168 meta-atoms. The algorithm calculates the directivity $D(\theta _r)$ for this initial configuration and compares it to the ideal directivity $D_\textrm{ideal}(\theta _r)$, which corresponds to a 1D array of 168 meta-atoms with unit amplitude and uniformly distributed phase values between 0$^{\circ }$ and 360$^{\circ }$. The key challenges arise from the simultaneous optimization of all 168 meta-atoms, resulting in a high-dimensional search space. To address this challenge, the optimization process begins with a reduced search domain, where only a small subset of meta-atoms is optimized, and then repeated periodically across the array. As the optimization progresses, the number of variables (i.e., the value of *i*) is gradually increased (with *i* = 8, 16, 24, 48, 56, 168) while using the current solution as the initial guess for the next iteration.

The GA uses MATLAB’s ‘gaoptimset’ to minimize the FOM by co-optimizing amplitude and phase values. The final solution, $\textbf{X}_\textrm{opt}$, is determined based on the minimum achieved FOM. Notably, the initially optimized index vector corresponding to $i = 8$, already accounts for the major contribution to the enhancement of the directivity, while subsequent optimization steps have a negligible contribution to the optimized value of $D(\theta _r)$, especially in our case where the phase modulation range is quite limited. Our computer, equipped with a 12th Gen Intel^®^ Core^TM^ i7-1260P processor, took approximately 6 minutes to optimize the first 8 meta-atoms over 400 generations. The first 150 generations contributed most significantly to the enhancement of the steered beam, taking approximately 3 minutes. This optimization time increased to 11 minutes during progressive full-array optimization, which involved 168 meta-atoms optimized in sequential order of 8, 16, 32, 64, and 168, with each stage undergoing 150 generations. This phase optimization approach can be further extended for the initial phase optimization of 2D beam steering (e.g., an $8 \times 8$ meta-atom array), whereas full-array optimization in scenarios such as $168 \times 168$ meta-atom arrays could be highly compute intensive. Furthermore, by employing a storage system that contains precalculated optimal phase profiles for specific beam steering angles, the need for recomputation in practical applications can be effectively avoided. On the other hand, for real-time optimization without relying on additional memory-based devices, one can implement gradient-based optimization, as it is faster and more efficient. However, such approaches carry the risk of convergence to local minima, which may limit performance in complex situations, such as when dealing with 2D arrays.

**Fig. 6:**
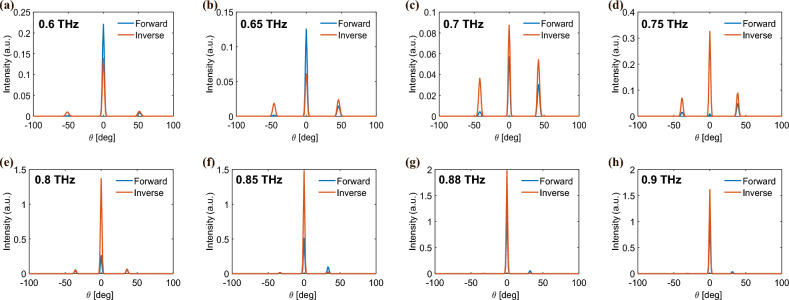
Simulated far-field intensity for a 1D phase gradient comprising 168 meta-atoms with a phase-gradient length of 8$P_x$. The forward (blue) and inversely (orange) designed intensity is plotted in a frequency range of 0.6 THz (a) to 0.9 THz (h).

The 1D-optimized index significantly enhances the directivity of the steered beam, which has been verified through CST simulations. However, this increase in directivity is not constant throughout the frequency spectrum and rather limited only to the lower frequency region, as shown in Fig. 6. Additionally, this technique is most effective at suppressing undesired lobes (transmitted beam along $m = -1$ and $m = 0$) only when the phase modulation range is large^39,40^. In our case the phase modulation range is limited, resulting in a beam steering performance similar to that of 2-bit binary coding metasurfaces. However, the simulation results demonstrate an increase in the directivity of the desired steered beam, proving to be an alternative method to realize efficient beam steering with a smaller phase modulation range.

Finally, note that it is possible to optimize the metasurface response for multiple frequencies separately. Nevertheless, in general, the resulting optimal phase profiles for each frequency will be incompatible with each other. Solving this problem would require increasing the number of degrees of freedom in the system by adding more reconfigurable elements to the unit cell.

## Discussion

Utilizing the continuously tunable phase-shift enabled by VO$_2$-patch incorporated meta-atom resonators, we presented an electrically-reconfigurable metasurface that can steer the co-polarized transmitted beam within a broad angular range, from -56$^{\circ }$ to +56$^{\circ }$. Additionally, we demonstrated that through the proper distribution of co-varying phase and amplitude of meta-atom resonators, the transmission efficiency of the desired steering beam can be significantly improved compared to forward-designed metasurfaces and coding metasurfaces. Moreover, we applied the inverse design technique to suppress the sidelobes and enhance the power concentrated in the main lobe. The proposed design is not limited to CSRR meta-atoms, and enables applications which are currently limited due to low available phase modulation ranges. It is also possible to demonstrate beam-steering in 2D by spatially controlling the conductivity of VO$_2$ along a 2D array. Our design can be used for ultra-fast beam-steering applications such as THz spectroscopy, ultra-fast imaging and LIDAR.

## Methods

We used the commercially available CST Studio software for the full-wave electromagnetic simulations of the meta-atoms and the full 2-dimensional antenna arrays. CST built-in functions were used to generate the illuminating Gaussian beam. The co-varying amplitude and phase obtained in CST were processed in MATLAB. The resulting phase and amplitude from inverse design optimization were used to analytically determine the directivity and efficiency of the steered beam.

## Data Availability

The datasets generated during and/or analysed during the current study are available from the corresponding authors on reasonable request.
